# Supplementary material for: Duplexed CeTEAM drug biosensors reveal determinants of PARP inhibitor selectivity in cells
Source: J Biol Chem. 2025 Feb 26;301(4):108361. doi: 10.1016/j.jbc.2025.108361 (PMC11986510; doi:10.1016/j.jbc.2025.108361)
Supplement: Supplementary Figures and Tables [file mmc1.pdf]

Supporting information

Duplexed CeTEAM drug biosensors reveal determinants of PARP inhibitor selectivity in cells

Maria J. Pires[1], Seher Alam[1], Alen Lovric[1], Emanuele Fabbrizi[2], Dante Rotili[3,4], Mikael Altun[5,*], Nicholas C.K. Valerie[5,*]

[1]Division of Clinical Physiology, Department of Laboratory Medicine, Karolinska Institutet, Karolinska University Hospital; Huddinge, SE-141 52, Sweden.
[2]Department of Chemistry and Technology of Drugs, Sapienza University of Rome; 00185 Roma RM, Italy.
[3]Department of Science, "Roma Tre" University, Viale G. Marconi 446, 00146 Rome, Italy.
[4]INBB - Biostructures and Biosystems National Institute, Via dei Carpegna 19, 00165 Rome, Italy.
[5]Science for Life Laboratory, Division of Clinical Physiology, Department of Laboratory Medicine, Karolinska Institutet, Karolinska University Hospital; Huddinge, SE-141 52, Sweden.

*Corresponding authors: nicholas.valerie@ki.se, mikael.altun@ki.se

Table of Contents

Supplementary tables 3

Table S1. Oligo annealing primers, plasmids and qPCR primers 3

Table S2. Screening compound details from the SciLifeLab Compound Center. 4

Supplementary figures 5

Supplementary Figure 1. Olaparib binding to PARP1 and PARP2 by cellular thermal shift assay (CETSA). 5

Supplementary Figure 2. PARPi binding to PARP1 L713F and PARP2 L269A by CETSA. 6

Supplementary Figure 3. Concentration-dependency of non-PARPi stabilization of PARP1 and PARP2 drug biosensors 7

Supplementary Figure 4. Bisindolylmaleimide I and Go 6983 are autofluorescent assay artefacts. 8

Supplementary Figure 5. Full dose-response curves for PARPi stabilization of PARP1 L713F-GFP and PARP2 L269A-mCherry in dual biosensor cells... 9

Supplementary Figure 6. Evaluation of additional PARPi on PARP1 L713F-GFP and PARP2 L269A-mCherry protein levels by western blot.. 10

Supplementary Figure 7. Cell cycle profile dynamics of tested PARPi. 11

Supplementary Figure 8. 2D multidimensional scaling (MDS) of PARPi structures........... 12

Supplementary Figure 9. Influence of HPF1 depletion on PARPi cell cycle dynamics. 13

Supplementary Figure 10. Time course of talazoparib and AZD5305 binding to PARP1 L713F-GFP and PARP2 L269A-mCherry in dual biosensor cells.. 14

Supplementary tables

Table S1. Oligo annealing primers, plasmids and qPCR primers

**Oligo annealing primers**	*Sequence (5'→ 3')*
shHPF1#1 F	CCGGGCTTGGTTGTTCCAGTAGATACTCGAG TATCTACTGGAACAACCAAGCTTTTTG
shHPF1#1 R	AATTCAAAAAGCTTGGTTGTTCCAGTAGATA CTCGAGTATCTACTGGAACAACCAAGC
shHPF1#2 F	CCGGGTGAAGAACTTGATCCTGAAACTCGA GTTTCAGGATCAAGTTCTTCACTTTTTG
shHPF1#2 R	AATTCAAAAAGTGAAGAACTTGATCCTGAA ACTCGAGTTTCAGGATCAAGTTCTTCAC
shHPF1#3 F	CCGGGCAAGTGATGAGGAGAGACTACTCGA GTAGTCTCTCCTCATCACTTGCTTTTTG
shHPF1#3 R	AATTCAAAAAGCAAGTGATGAGGAGAGACT ACTCGAGTAGTCTCTCCTCATCACTTGC
**Plasmids**	*Source*
pENTR1a-PARP1 L713F-GFP	Valerie., *et al.*[1]
pENTR1a-PARP1 WT-GFP	Valerie., *et al.*[1]
pENTR4-mCherry-PARP1 WT	This work
pLenti CMV Blast_PARP1 L713F-GFP	This work
pINDUCER20-PARP1 L713F-GFP	Valerie., *et al.*[1]
pINDUCER20-PARP1 WT-GFP	Valerie., *et al.*[1]
Ef1a-Tta3G-P2A-Blast_PARP1 WT_mCherry	This work
pLenti CMV Puro_PARP1 WT-mCherry	This work
pET28-PARP2 L269A	Langelier, M.-F., *et al.*[2]
TOPO-PARP2 L269A	Valerie., *et al.*[1]
pET28-PARP2 WT	Langelier, M.-F., *et al.*[2]
TOPO-PARP2 WT	This work
pENTR1a-PARP2 L269A-mCherry	This work
pINDUCER20-PARP2 WT-GFP	This work
pLenti CMV Hygro_PARP2 WT-mCherry	This work
pLenti CMV Hygro_PARP2 L269A-mCherry	This work
prSITEP_Puro_akaLuc-shHPF1#1	This work
prSITEP_Puro_akaLuc-shHPF1#2	This work
prSITEP_Puro_akaLuc-shHPF1#3	This work
prSITEP_Puro_akaLuc-shNT	This work
**qPCR primers**	*Sequence (5'→ 3')*
HPF1 F	AGAAAGTTGTGACAAAGACC
HPF1 R	CATCATTTCCTGAATGGGAG
β-actin F	CCTGGCACCCAGCACAAT
β-actin R	GGGCCGGACTCGTCATACT

Table S2. Screening compound details from the SciLifeLab Compound Center.

Compound	Compound ID	Batch number	Stock (mM)	Volume (nL)
Linifanib (ABT-869)	CBK169041	BJ1886001	10	100
Olaparib (AZD2281)	CBK277996	BJ1886025	10	100
Paclitaxel	CBK011621	BJ1886057	10	100
PF-3758309	CBK293899	BJ1886257	10	100
Veliparib (ABT-888)	CBK277926	BJ1886002	10	100
AT9283	CBK277923	BJ1886053	10	100
Niraparib (R-enantiomer)	CBK278031	BJ1886189	10	100
Rucaparib (phosphate)	CBK277950G	BJ1886038	10	100
5-Azacytidine	CBK041875	BJ1886134	10	100
Decitabine	CBK201329	BJ1886078	10	100
Tankyrase-IN-2	CBK506899	DO8144443	10	100
Decitabine	CBK201329	DO8144447	10	100
BYK204165	CBK290351	DO8144458	10	100
Olaparib (AZD2281)	CBK277996	DO8144485	10	100
AG14361	CBK290655	DO8144498	10	100
Bisindolylmaleimide I	CBK040989	DO8144499	10	100
E7449	CBK308723	DO8144504	10	100
AZ960	CBK288327	DO8144511	10	100
Go 6983	CBK290912	DO8144531	10	100
INO-1001	CBK506935	DO8144542	10	100
PJ34 (hydrochloride)	CBK308811C	DO8144559	10	100
AZD-2461	CBK290993	DO8144576	10	100
Rucaparib	CBK277950	DO8144602	10	100
Niraparib (tosylate)	CBK278031G	DO8144605	10	100
Rucaparib (Camsylate)	CBK277950H	DO8144641	10	100
Fluzoparib	CBK506988	DO8144643	10	100
1,5-Isoquinolinediol	CBK507027	DO8144711	10	100
Talazoparib	CBK309483	DO8144726	10	100
Pamiparib	CBK506999	DO8144660	10	100
A-966492	CBK507022	DO8144701	10	100
Senaparib	CBK506721	DO8144123	10	100
Veliparib (dihydrochloride)	CBK277926C	DO8144731	10	100
ACY-1083	CBK506771	DO8144206	10	100
A-395	CBK506730	DO8144137	10	100
Veliparib (ABT-888)	CBK277926	DO8144161	10	100
AZ9482	CBK506867	DO8144391	10	100
Niraparib (R-enantiomer)	CBK290547	DO8144329	10	100
KDM5-C70	CBK506855	DO8144369	10	100
Rucaparib (phosphate)	CBK277950G	DO8144062	10	100
Mefuparib (hydrochloride)	CBK506689C	DO8144061	10	100
Niraparib (R-enantiomer)	CBK278031	DO8144020	10	100
NMS-P118	CBK506686	DO8144053	10	100
Niraprib (tosylate)	CBK278031	DO8144101	10	100
Venadaparib	CBK506696	DO8144071	10	100
Niraparib (hydrochloride)	CBK278031C	DO8144089	10	100
Talazoparib tosylate	CBK309483G	DO8144102	10	100
AZD5305	CBK506718	DO8144116	10	100

Supplementary figures

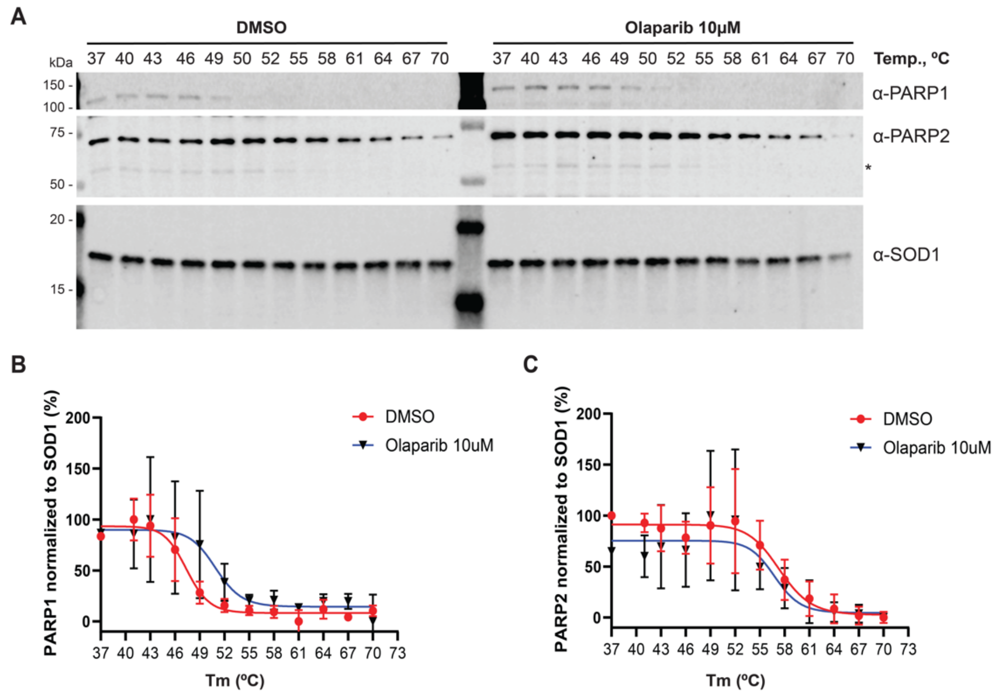

Supplementary Figure 1. Olaparib binding to PARP1 and PARP2 by cellular thermal shift assay (CETSA). A. Western blot analysis was employed to evaluate the thermal stability of PARP1 and PARP2 by CETSA after DMSO or 10 µM olaparib and was performed across a temperature range of 37ºC to 70ºC in 3ºC increments. Proteins were detected using specific antibodies against PARP1 and PARP2, with SOD1 serving as the loading control to ensure consistent protein quantification across samples. Asterisk (*) denotes a non-specific band from the PARP2 antibody. **B.** Melting profiles of PARP1 after DMSO (red) or 10 µM olaparib treatment (black/blue). Protein levels are normalized to SOD1 and set relative to 37ºC abundance (percent). **C.** Melting profiles of PARP2, as in **B**. Means from n=3 replicates ± SD with lines-of-best-fit shown in both instances.

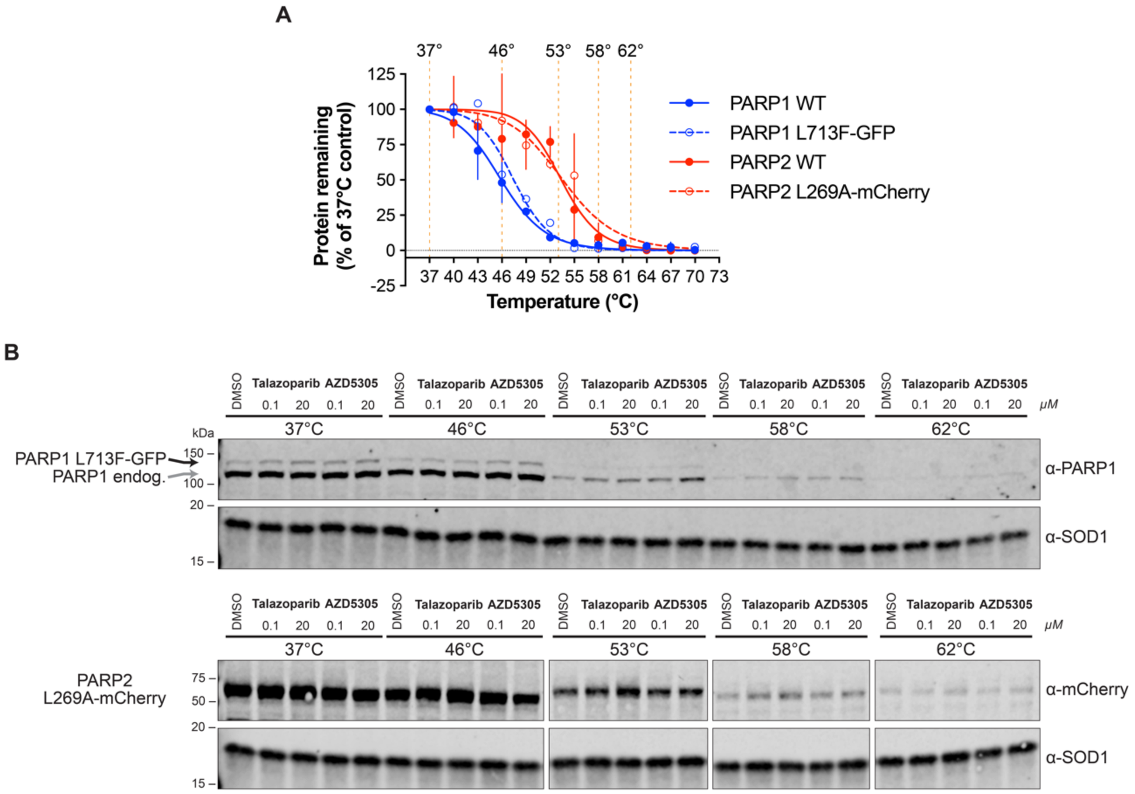

Supplementary Figure 2. PARPi binding to PARP1 L713F and PARP2 L269A by CETSA. A. CETSA melt curves from U-2 OS lysates comparing CeTEAM biosensors (open circles) to endogenous, WT PARP1 and PARP2 (filled circles). Cherry-picked temperatures for testing PARPi binding at different points of the melt curve are demarcated in orange. Protein abundance is normalized to SOD1 and set relative to the 37°C control. Means ± range from n=2 experiments are shown. **B**. CETSA stabilization of PARP1 L713F-GFP and PARP2 L269A-mCherry by low (0.1 µM) and high (20 µM) concentrations of talazoparib or AZD5305 in U-2 OS cell lysates. PARPi were incubated for 25 min at room temperature and subsequently heat pulsed at 37, 46, 53 and 58°C. PARP1 L713F-GFP (black arrow) and endogenous PARP1 (grey arrow) are probed with an α-PARP1 antibody. DMSO controls are included at each temperature for comparison and thermostable SOD1 is included as a loading control. Representative blots from two independent experiments are shown. PARP2 L269A-mCherry samples (lower panel) were misloaded and rearranged so that the temperature gradient is in the correct order.

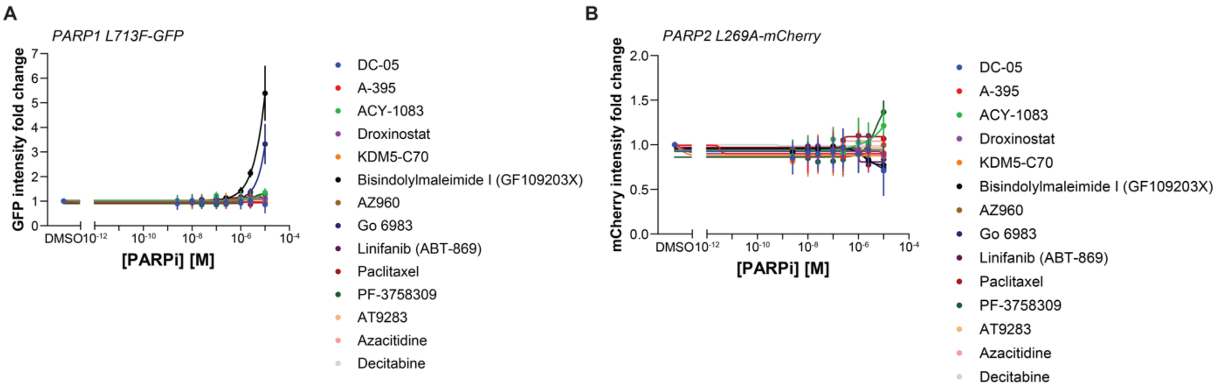

Supplementary Figure 3. Concentration-dependency of non-PARPi stabilization of PARP1 and PARP2 drug biosensors. A. PARP1 L713F-GFP intensity fold change after non-PARPi drug gradients for 16 hours. Data normalized to DMSO control. Means from n=3 experiments ± SD and lines-of-best-fit are shown. **B.** PARP2 L269A-mCherry intensity fold change, as in **A**.

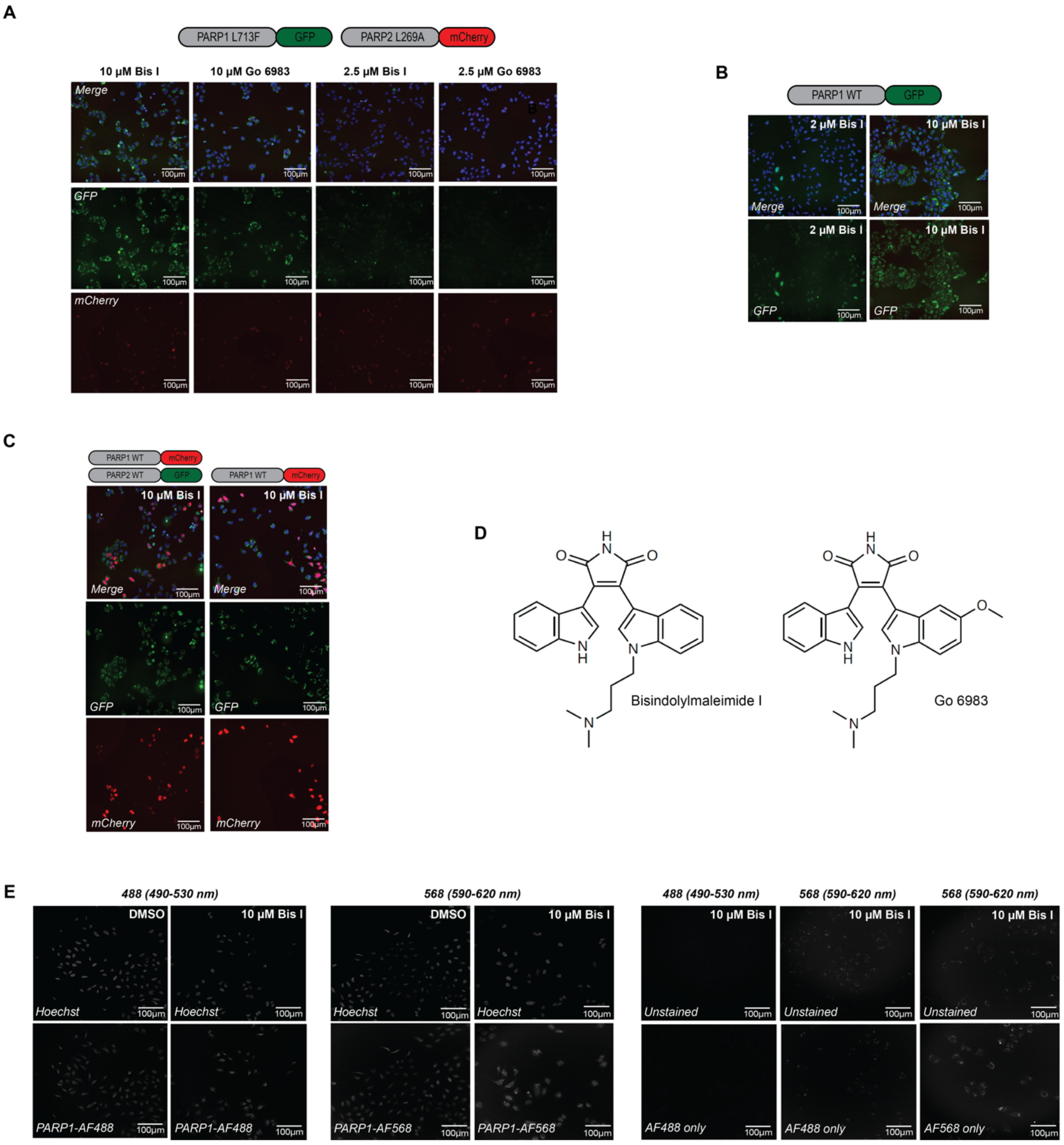

Supplementary Figure 4. Bisindolylmaleimide I and Go 6983 are autofluorescent assay artefacts. **A.** Live cell fluorescent microscopy of Bisindolylmaleimide I (Bis I) and Go 6983 effects on subcellular localization of PARP1 L713F-GFP and PARP2 L269A-mCherry at 2.5 or 10 µM. Distinct perinuclear accumulation of L713F-GFP is seen but not for L269A-mCherry. Scale bars = 100 µm. **B.** Live cell fluorescent microscopy of Bis I and Go 6983 effects on subcellular localization of PARP1 WT-GFP. Scale bars = 100 µm. **C.** Live-cell fluorescent microscopy of 10 µM Bis I effects on distribution of PARP1 WT-mCherry/PARP2 WT-GFP (left) or PARP1 WT-mCherry alone (right). Scale bars = 100 µm. **D.** Structures of Bisindolylmaleimide I and Go 6983. **E.** Immunofluorescence confocal microscopy assessing endogenous PARP1 localization in U-2 OS cells treated with DMSO or Bisindolylmaleimide I, using an anti-PARP1 primary antibody and Alexa Fluor 488 or Alexa Fluor 568 as secondary antibodies. Endogenous PARP1 was detected in spectrally distinct 488 (490-530 nm) or 568 (590-620) filters. Unstained cells or those with secondary antibody only were also treated with Bis I for reference. Scale bars = 100 µm.

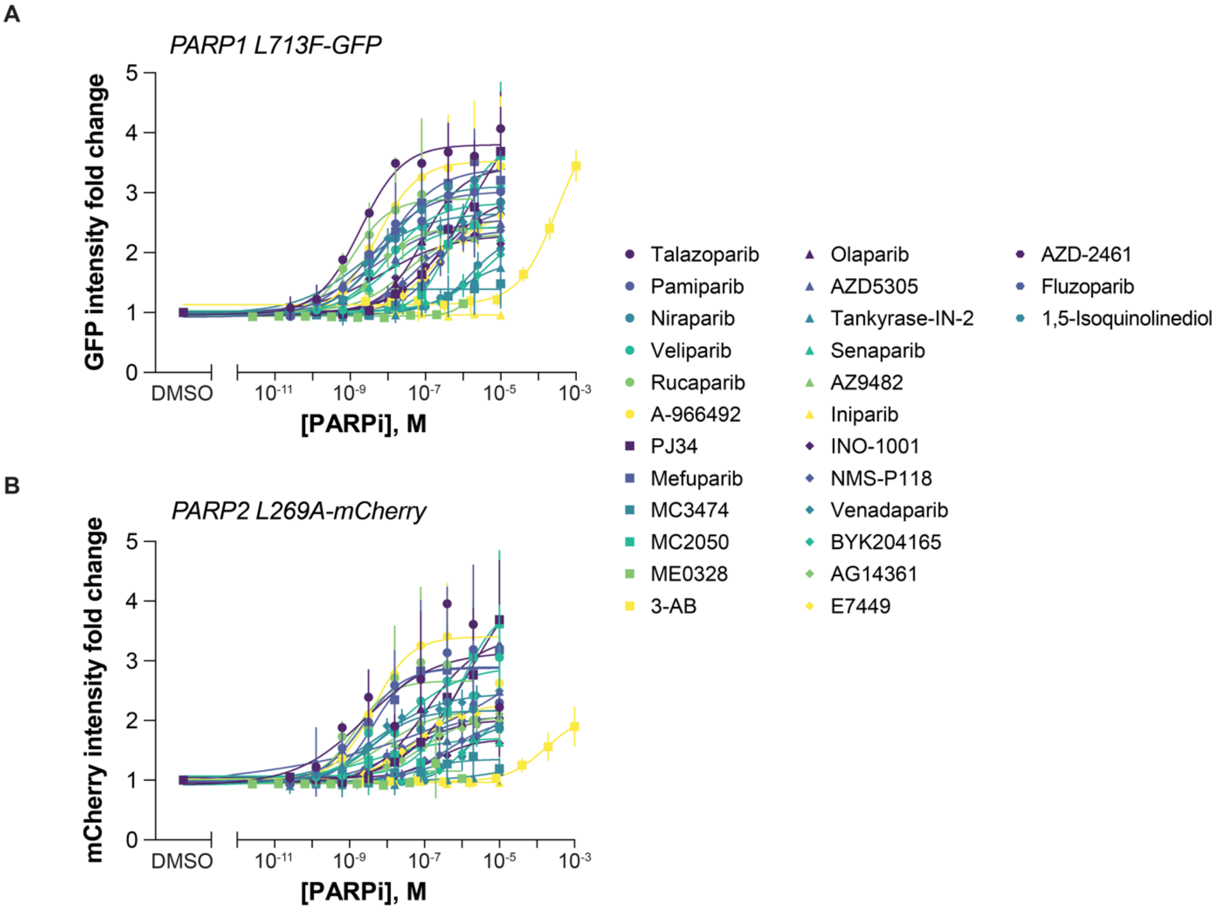

Supplementary Figure 5. Full dose-response curves for PARPi stabilization of PARP1 L713F-GFP and PARP2 L269A-mCherry in dual biosensor cells. A. Live-cell L713F-GFP fold change by fluorescence microscopy following a concentration gradient with the indicated PARPi for 24 hours and normalization to DMSO controls. Means ± SD (n=3) with lines-of-best-fit shown. **B.** Live-cell L269A-mCherry fold change by fluorescence microscopy, as in **A**. Means ± SD (n=3) with lines-of-best-fit shown.

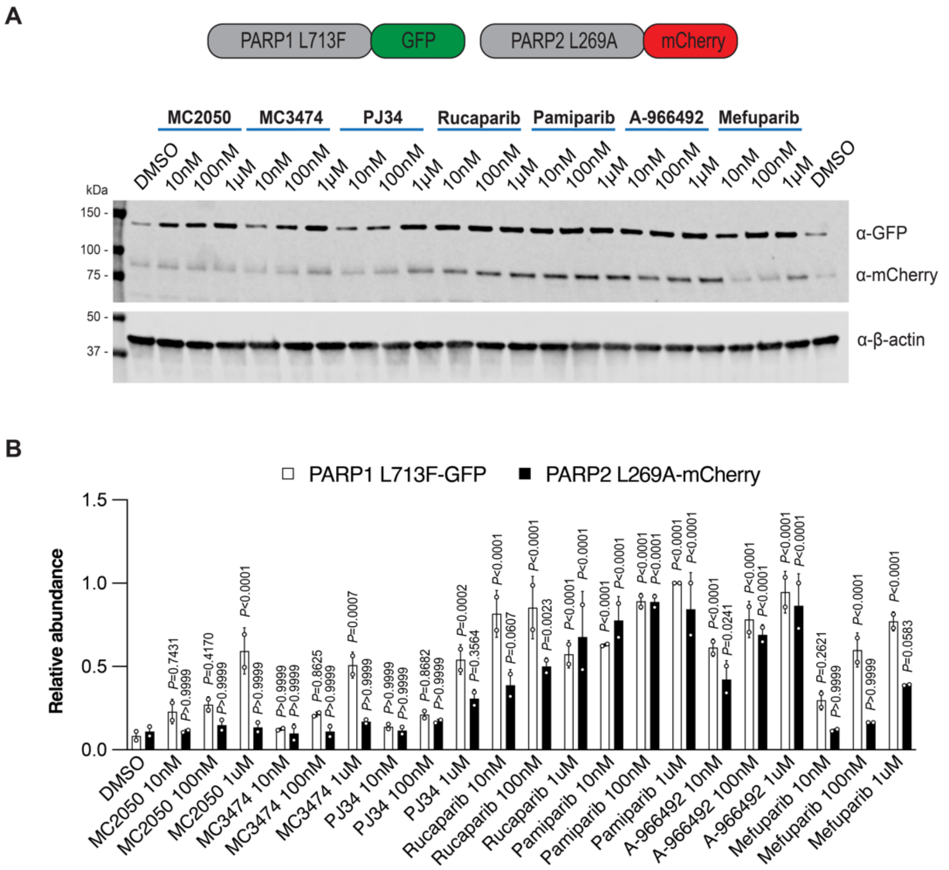

Supplementary Figure 6. Evaluation of additional PARPi on PARP1 L713F-GFP and PARP2 L269A-mCherry protein levels by Western blot. A. A representative western blot of PARP1 L713F-GFP and PARP2 L269A-mCherry abundance 24 hours after MC2050, MC3474, PJ34, rucaparib, pamiparib, A-966492, or mefuparib treatment at the indicated concentrations. **B.** Densitometric quantification of PARP1 L713F-GFP (green) and PARP2 L269A-mCherry (red) protein levels relative to β-actin and normalized to the highest value recorded within the experiment to ensure comparability across treatments. Means ± SD are shown from n=2 experiments. Statistical significance was determined by ordinary two-way ANOVA with Dunnett's post-test, comparing each treatment to the relevant DMSO control ($F_{Interaction}$[DFn, DFd]: 3.585[21, 44], P=0.0002; F_{Row}[DFn, DFd]: 35.27[21, 44], P<0.0001; F_{Column}[DFn, DFd]: 63.68[1, 44], P<0.0001).

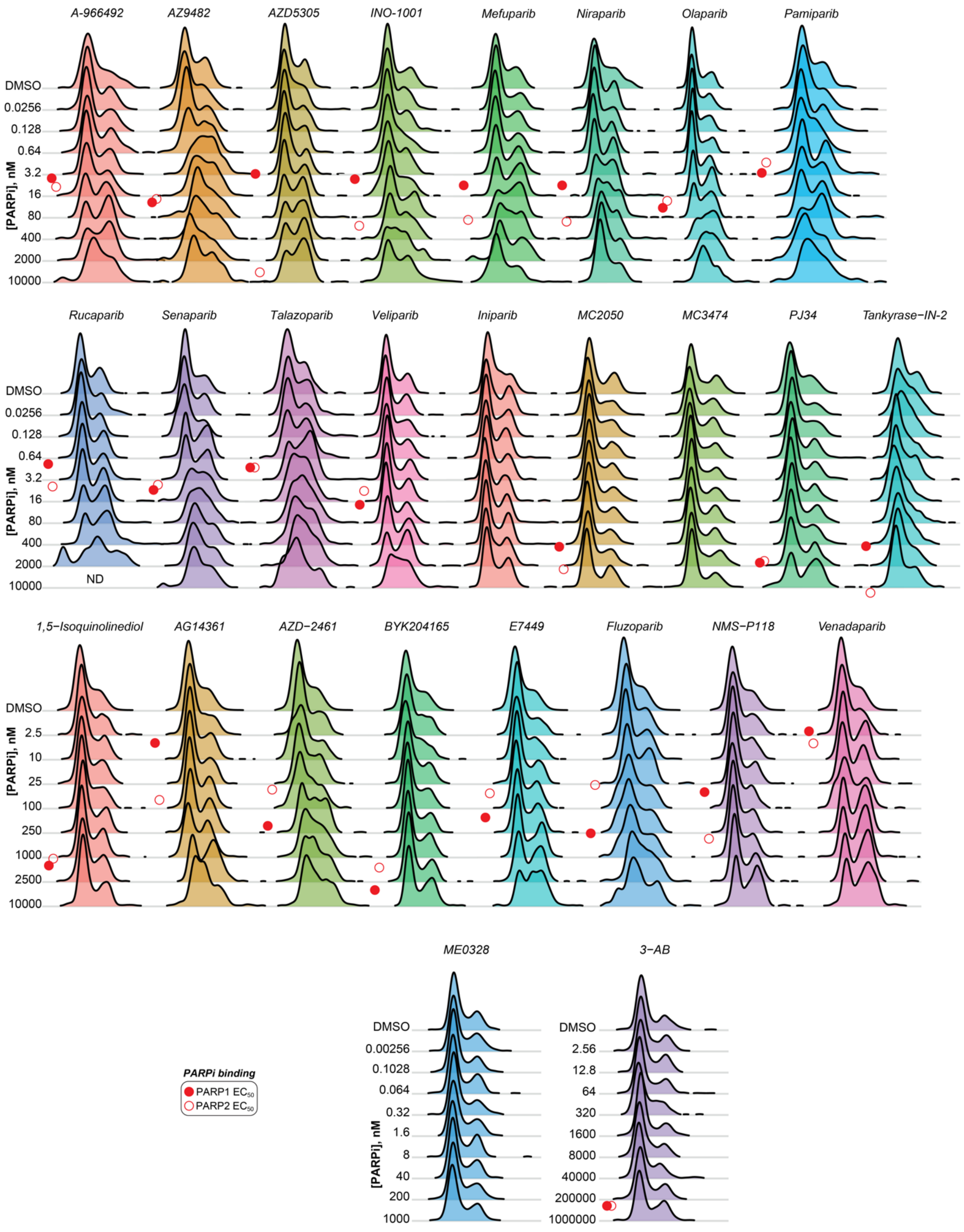

Supplementary Figure 7. Cell cycle profile dynamics of tested PARPi. Representative cell cycle profile dynamics from Hoechst intensity for PARPi concentration dependency, as shown by Ridgeline plot. Concentrations of PARPi are indicated on the lefthand side of the plots. Red circles – approximate stabilization EC_{50} values for PARP1 (closed) or PARP2 (open) biosensors.

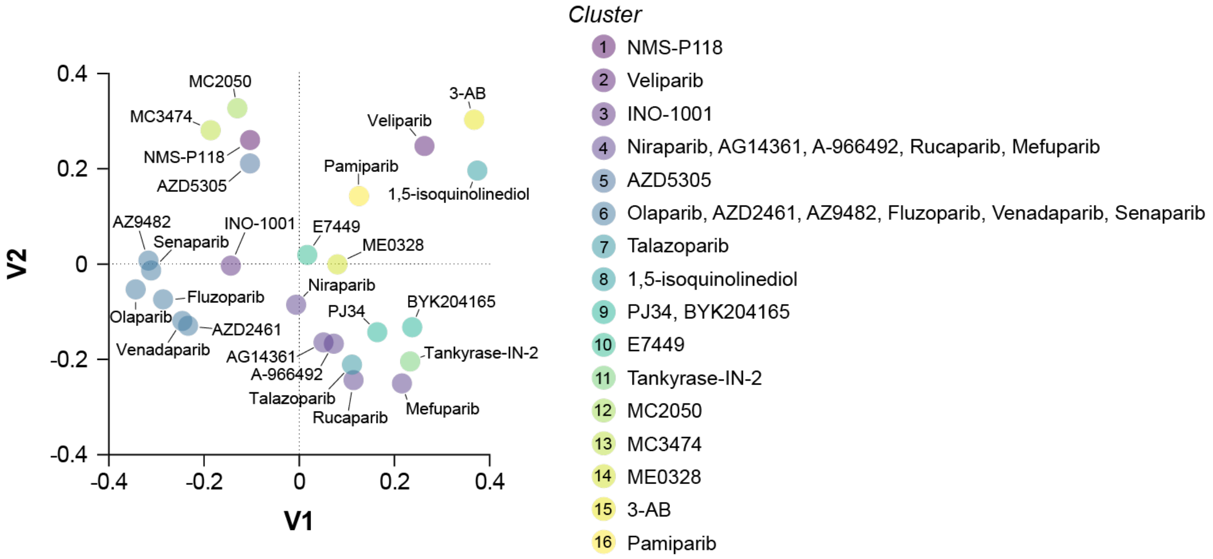

Supplementary Figure 8. 2D multidimensional scaling (MDS) of PARPi structures. Structural similarity clustering and MDS plotting was performed with the ChemMine Tools online server with a Tanimoto coefficient cutoff of 0.4 for determining clusters. Clusters are defined by number and associated color label with PARPi falling into each cluster labelled.

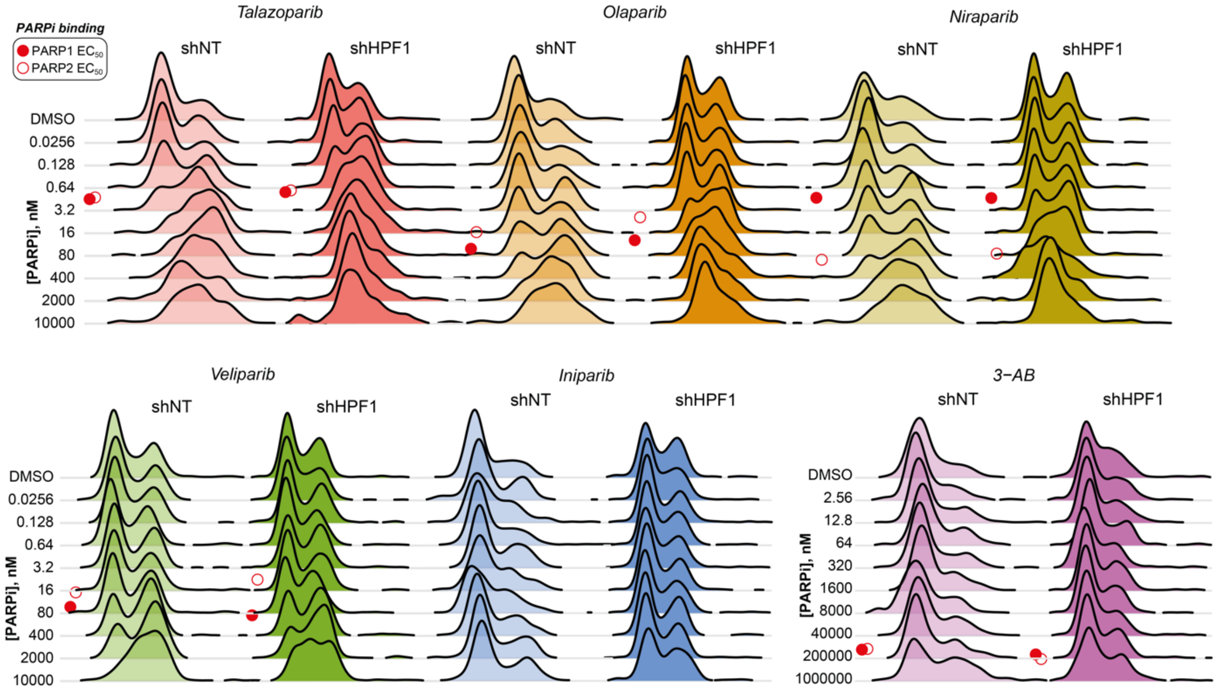

Supplementary Figure 9. Influence of HPF1 depletion on PARPi cell cycle dynamics. Representative cell cycle profile dynamics from Hoechst intensity of shNT or shHPF1 cells after 24-hour PARPi at different concentrations, as shown by Ridgeline plot. 3-AB had a concentration range of 2.56 to 1,000,000 nM, all others – 0.0256 to 10,000 nM. Red circles – approximate stabilization EC_{50} values for PARP1 (closed) or PARP2 (open) biosensors.

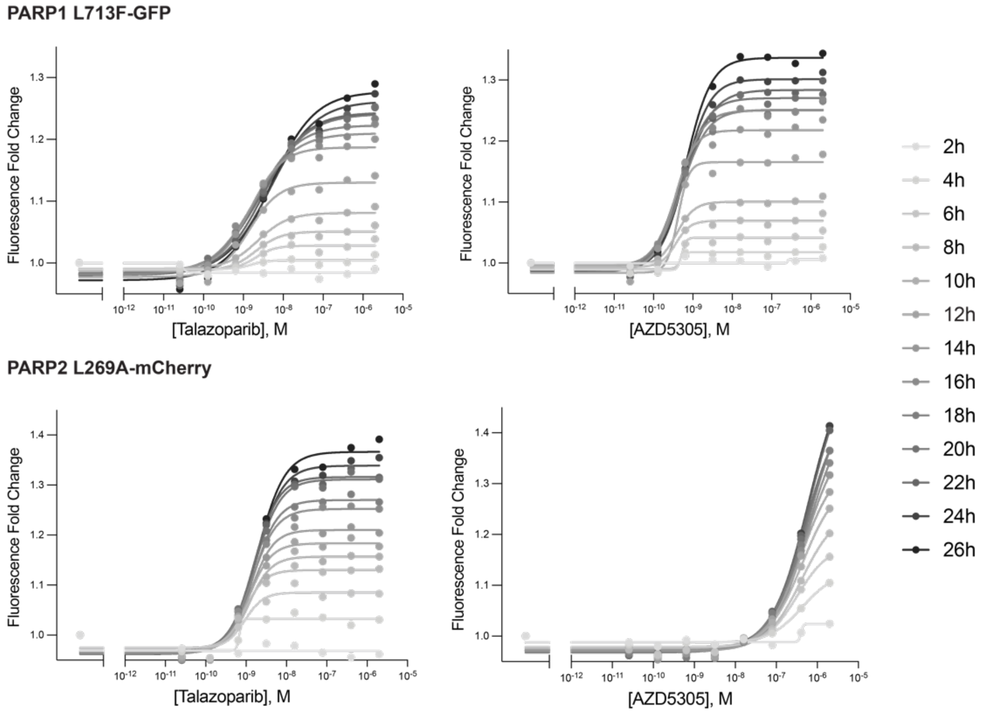

Supplementary Figure 10. Time course of talazoparib and AZD5305 binding to PARP1 L713F-GFP and PARP2 L269A-mCherry in dual biosensor cells. Representative live-cell fluorescence signals of PARP1 L713F-GFP and PARP2 L269A-mCherry in response to the indicated concentration-gradients of talazoparib and AZD5305 relative to DMSO control for each time point. Fluorescence intensity was measured every 2h for 24 hours and lines-of-best-fit are shown. A representative of three independent experiments is shown.
